# Long-acting stimulants for treatment of attention-deficit/hyperactivity disorder: a focus on extended-release formulations and the prodrug lisdexamfetamine dimesylate to address continuing clinical challenges

**DOI:** 10.1007/s12402-013-0106-x

**Published:** 2013-04-06

**Authors:** Frank A. López, Jacques R. Leroux

**Affiliations:** 1 Children’s Developmental Center, Winter Park, FL USA; 2 Hôpital Rivière-des-Prairies, 7070 boul Perras, Montreal, QC H1E 1A4 Canada

**Keywords:** Attention-deficit hyperactivity disorder, Central nervous system stimulants, Amphetamines, Methylphenidate, Treatment efficacy, Safety

## Abstract

Individuals with attention-deficit/hyperactivity disorder (ADHD) show pervasive impairments across family, peer, and school or work functioning that may extend throughout the day. Psychostimulants are highly effective medications for the treatment of ADHD, and the development of long-acting stimulant formulations has greatly expanded the treatment options for individuals with ADHD. Strategies for the formulation of long-acting stimulants include the combination of immediate-release and delayed-release beads, and an osmotic-release oral system. A recent development is the availability of the first prodrug stimulant, lisdexamfetamine dimesylate (LDX). LDX itself is inactive but is cleaved enzymatically, primarily in the bloodstream, to release *d*-amphetamine (*d*-AMP). Several clinical trials have demonstrated that long-acting stimulants are effective in reducing ADHD symptoms compared with placebo. Analog classroom and simulated adult workplace environment studies have shown that long-acting stimulants produce symptom reduction for at least 12 h. Long-acting stimulants exhibit similar tolerability and safety profiles to short-acting equivalents. While variations in gastric pH and motility can alter the availability and absorption of stimulants released from long-acting formulations, the systemic exposure to *d*-AMP following LDX administration is unlikely to be affected by gastrointestinal conditions. Long-acting formulations may also improve adherence and lower abuse potential compared with their short-acting counterparts. The development of long-acting stimulants provides physicians with an increased range of medication options to help tailor treatment for individuals with ADHD.

## Introduction

Attention-deficit/hyperactivity disorder (ADHD) is a common neurobehavioral disorder that is estimated to affect 5–12 % of children and persists into adulthood in more than half of cases (Biederman and Faraone 2005; Polanczyk et al. 2007). ADHD is characterized by the core symptoms of inattention, hyperactivity, and impulsivity (American Psychiatric Association 2000). In addition, individuals with ADHD exhibit functional impairments that include poor intrafamily interactions, low academic achievement and conduct problems in children and adolescents, and increased risk of lower educational attainment, behavior leading to arrests and traffic violations, unemployment, and divorce in adults (Able et al. 2007; Barkley et al. 2006; Biederman et al. 2006a; Kessler et al. 2006; Klassen et al. 2004; Sawyer et al. 2002).

Clinical guidelines for the treatment for ADHD generally recommend an individualized, multimodal plan which includes pharmacotherapy, behavioral, and educational interventions (American Academy of Pediatrics 2011; Canadian Attention Deficit Hyperactivity Disorder Resource Alliance (CADDRA) 2011; National Institute for Health and Clinical Excellence 2009; Pliszka 2007). For many years, short-acting formulations of the psychostimulants methylphenidate (MPH) and amphetamine (AMP) were the mainstay of ADHD pharmacotherapy. However, despite their well-documented efficacy, durations of action in the range 3–6 h posed significant challenges and limitations in their treatment for ADHD (Antshel et al. 2011). The requirement for repeated dosing during the day may cause embarrassment and stigma for the patient, difficulties associated with storing scheduled drugs, especially in a school environment, fragmented coverage, poor adherence, and the potential for the diversion of drug for non-medical use (Swanson 2003; Wolraich et al. 2001). In response to these challenges, long-acting psychostimulants were developed to relieve ADHD symptoms throughout the day without the need for repeat dosing and to improve adherence compared with short-acting agents (Adler and Nierenberg 2010; Christensen et al. 2010; Ramos-Quiroga et al. 2008; Spencer et al. 2011; van den Ban et al. 2010).

The variation in the pharmacokinetic properties of the different formulations of long-acting psychostimulant therapies is reflected in their pharmacodynamic properties including their onset, magnitude, and duration of symptom relief. Treatment strategies should be based on an understanding of the efficacy and safety profile of each formulation, paired with individual patient needs. Long-acting psychostimulants, as well as the non-stimulant atomoxetine, are recommended as first-line pharmacotherapies in many countries for the management of ADHD in children, adolescents, and adults (American Academy of Pediatrics 2011; Canadian Attention Deficit Hyperactivity Disorder Resource Alliance (CADDRA) 2011; National Institute for Health and Clinical Excellence 2009; Pliszka 2007). This article will review the different controlled-release and prodrug delivery systems of long-acting stimulants, and examine the impact of these formulations on their pharmacokinetics, efficacy, safety, and adherence.

## Long-acting stimulant formulations

**Fig. 1:**
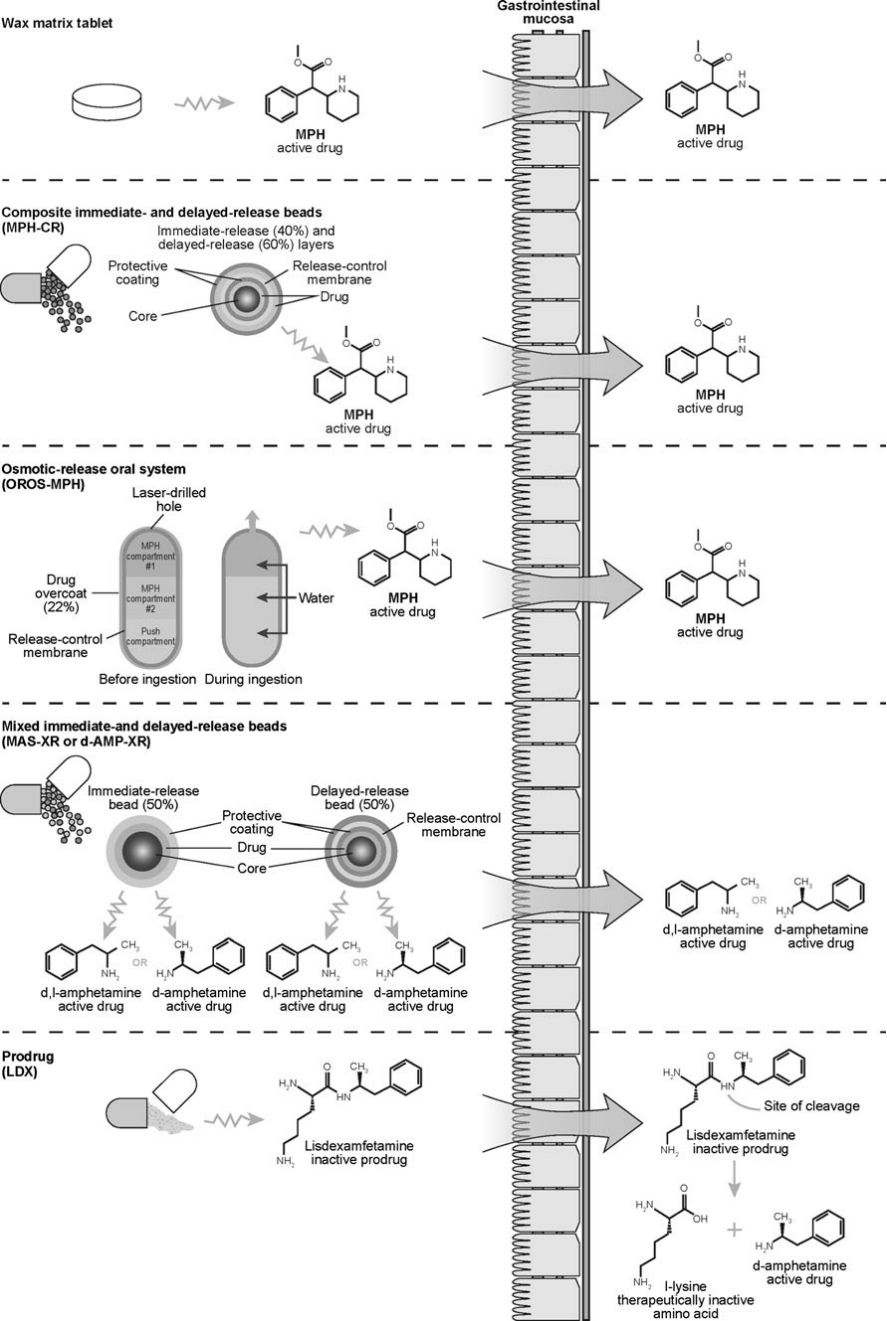
Delivery systems of long-acting psychostimulants used in the treatment for ADHD. Not shown are the delivery systems of MPH-SR and Novo-MPH ER-C. MPH-SR is an extended-release formulation in tablet form that uses a wax-based matrix to achieve prolonged release (Ermer et al. 2010b). The delivery mechanism of Novo-MPH ER-C has not been published (Canadian Attention Deficit Hyperactivity Disorder Resource Alliance (CADDRA)). *CR* controlled-release, *d*-AMP *d*-amphetamine, *LDX* lisdexamfetamine dimesylate, *MAS* mixed amphetamine salts, *MPH* methylphenidate, *SR* sustained-release, *XR* extended-release

The long-acting psychostimulants that have been approved for the treatment for ADHD can be categorized according to the technology that has been utilized to extend or delay the release of the active agent (Fig. 1) (Table 1). The first generation of long-acting stimulants included sustained-release formulations of MPH (MPH-SR) that utilized a wax-matrix-based technology to deliver a single, prolonged pulse of MPH. With a duration of action of up to 8 h, some authors consider these preparations to be intermediate- rather than long-acting (Dopheide 2009), and their efficacy may be inferior to multiple-dose regimens of the immediate-release formulations (Swanson and Volkow 2009). The flat (zero-order) drug delivery profile of MPH-SR may account for the development of acute drug tolerance in response to exposure to relatively high drug levels over a prolonged period (Swanson 2003).

Several controlled-release (CR) stimulant formulations were developed that combined a rapid onset of action with extended coverage throughout the day. One strategy for the biphasic delivery of stimulants is to mix beads with different drug release profiles. CR capsules contain beads that incorporate MPH (MPH-CR) or *d*-AMP (AMP-CR) with distinct immediate- and delayed-release profiles (Fig. 1). CR-mixed AMP salts comprise equal proportions of four AMP salts, *d*-AMP saccharate, *d*,*l*-AMP aspartate monohydrate, *d*-AMP sulfate, and *d*,*l*-AMP sulfate. Each capsule contains immediate-release and enteric-coated delayed-release beads in a 1:1 ratio (Shire Canada Inc.; Tulloch et al. 2002). Several stimulant drugs are based on combinations of beads with immediate- and extended-release profiles, but the proportions of the total dose of active ingredient in the two phases of delivery vary.

An alternative technology designed to deliver the controlled and biphasic delivery of stimulant medication is the osmotic-release oral system methylphenidate (OROS-MPH). OROS-MPH utilizes osmotic pressure to control the rate of delivery of the active ingredient, racemic MPH. Each capsule consists of a three-compartment core that is enclosed by a semipermeable membrane that, in turn, is surrounded by a drug overcoat (Fig. 1). After ingestion, the drug overcoat provides immediate release of MPH (22 % of the dose) (McBurnett and Starr 2011). Aqueous fluid enters the osmotic pump compartment from the gastrointestinal tract and delivers the remaining dose at a first-order rate from the core through a laser-drilled exit port (Janssen Inc.; Swanson et al. 2004). A concentration gradient exists between the two drug compartments, which also modifies the rate of drug release. OROS-MPH has a smooth ascending plasma concentration profile, which is thought to minimize the development of acute tolerance and maintain full efficacy across the day (Swanson et al. 2003).

Lisdexamfetamine dimesylate (LDX) is the first prodrug stimulant. Rather than utilizing a mechanical or physical mechanism to achieve a prolonged duration of action, LDX is a prodrug in which *d*-AMP is covalently bound to the amino acid lysine. LDX itself is therapeutically inactive but, after oral administration, enzymatic hydrolysis of LDX releases the therapeutically active moiety *d*-AMP (Pennick 2010). The rate of enzymatic conversion controls the rate at which *d*-AMP becomes available. The resulting pharmacokinetic profile is dose-proportional and monophasic and reflects the gradual conversion of LDX to *d*-AMP over the post-dose period (Boellner et al. 2010). As enzymatic hydrolysis occurs mostly in the bloodstream (Pennick 2010), the metabolic conversion of LDX to *d*-AMP is unlikely to be affected by variations in gastric pH or gastrointestinal transit time (Ermer et al. 2010b; Haffey et al. 2009; Krishnan and Zhang 2008). Thus, pharmacokinetic studies have shown that the rate of *d*-AMP absorption and metabolism is more consistent and predictable between and within individuals with LDX than with extended-release mixed amphetamine salts (MAS-XR) (Biederman et al. 2007a; Ermer et al. 2010a). Furthermore, the rate of *d*-AMP delivery following administration of LDX is reported to be unaffected by concurrent administration of the acid-suppressing drug omeprazole, whereas a shortened time to peak concentration of *d*-AMP was observed when MAS-XR was taken with omeprazole (Haffey et al. 2009) (Table 1).

**Table 1:** Summary of mean (SD) pharmacokinetic parameters observed for selected long-acting stimulants

Delivery system technology	Medication, dose (mg)	Patients	*C*_max_ (ng/mL)^a^ Mean (SD)	AUC_0–inf_ (ng·h/mL) Mean (SD)	*T*_max_ (h)^a,b^	*t*_1/2_ (h) Mean (SD)
Wax-based matrix	MPH-SR (Novartis Pharmaceuticals Canada Inc.) 20 mg	Children/adults		Relative bioavailability to IR-MPH: Children: 105 % Adults: 101 %^c^	4.7 (range, 1.3 to 8.2)^d^ in children	Children: 2.4 Adults: 2.1
Composite IR and DR beads	MPH-CR (Quinn et al. 2007) Mean (SD) dose 38.6 (17.7) mg/day	Children with ADHD (*n* = 14)	12.1 (5.76)	155.1 (71.16)	4.0 (2.61)	5.1 (1.47)
Mixed IR and DR beads	MAS-XR (Shire Canada Inc.; McGough et al. 2003) 20 mg	Children with ADHD (*n* = 48)	40.1 (1.6)^g^	893.3 (62.2)^g^	7.1 (0.4)^g^	NA
Osmotic-release oral system	OROS-MPH (Janssen Inc.; Gonzalez et al. 2002) 18–72 mg	Healthy adults (18 mg; *n* = 36)	1: 2.1 (1.0) 2: 3.4 (1.2)	36.4 (13.5)	~6.0^b^	3.6 (0.7)
		Children with ADHD (36 mg; *n* = 7)	11.3 (2.6)	87.7 (18.2)^e^	8.1 (1.1)	
		Adolescents with ADHD (72 mg; *n* = 6)^f^	17.8 (4.5)	186 (33.9)	7.0 (1.8)	3.5 (0.5)
Prodrug	LDX (Shire Canada Inc.; Krishnan and Zhang 2008; Biederman et al. 2007a; Ermer et al. 2010b) 70 mg	Children with ADHD (*n* = 8) Healthy adults (*n* = 18)	155 (31.4)^h^ 69.3 (14.3)^h^	1,326 (285.8)^h,i^ 1,010 (314.2)^h^	4.5^b,h^ 3.8 (1.01)^h^	NA 9.7 ^h^ (1.96)

^a^Biphasic *C*_max_ and *T*_max_ reported when pertinent and available

^b^ *T*_max_ reported as median or time at which the mean *C*_max_ was observed in the noted cases, otherwise reported as mean (SD)

^c^Mean not available. Relative bioavailability cited

^d^Depicts range of values

^e^AUC_0–11.5_ is shown. AUC calculated from time 0 to 11.5 h post-dose

^f^72 mg OROS-MPH is not a recommended dose for children/adolescents

^g^Values are for *d*-AMP enantiomer

^h^Values are for *d*-AMP derived from LDX

^i^AUC_last_ is shown; AUC calculated from time 0 to time of the last quantifiable plasma drug concentration using the linear trapezoidal rule

*AUC* area under the plasma drug concentration:time curve, *CR* controlled-release, *C*_max_, maximum plasma drug concentration, *d*-AMP *d*-amphetamine, *DR* delayed-release, *GI* gastrointestinal, *IR* immediate-release, *LDX* lisdexamfetamine dimesylate, *MAS* mixed amphetamine salts, *MPH* methylphenidate, *NA* parameter not available, *OROS* osmotic-release oral system, *SR* sustained-release, *T*_max_ time to maximum plasma drug concentration, t_1/2_, terminal elimination half-life, *XR* extended-release

## Efficacy of long-acting stimulants

Clinical trial evidence supports the efficacy of long-acting stimulants. Tables 2 and 3 present a summary of short-term (≤13-weeks), randomized, controlled clinical efficacy trials of long-acting psychostimulants. The control of symptoms throughout the day and into the early evening is likely to be an important factor in the overall efficacy of ADHD pharmacotherapies (Coghill et al. 2008).

**Table 2:** Short-term (≤13-week), randomized, controlled clinical efficacy trials of long-acting methylphenidate-based stimulants in children and adults with ADHD

Author (year)	Participants (age, *N*)	Duration/design	Treatment (dose)	Primary outcome^a^
MPH-SR				
Pelham et al. (1987)	6.6–11 years Study 1: *N* = 13 Study 2: *N* = 9	Double-blind, randomized, placebo-controlled 7 weeks/crossover	MPH-SR: 20 mg/day IR-MPH: 10 mg BID Placebo	*ACTRS* MPH-SR: 3.4 (4.87) IR-MPH: 1.9 (2.00) Placebo: 4.6 (3.75) MPH-SR + IR-MPH average vs placebo (*p* = NS)
MPH-CR				
Weiss et al. (2007)	6.4–17.5 years *N* = 90	Double-blind, randomized, 5 weeks/crossover	MPH-CR: 10–60 mg/day IR-MPH: 10–60 mg/day	*CGI-I* MPH-CR: 2.3 (1.1) vs IR-MPH: 2.3 (1.3) (*p* = NS) *CPRS-R* MPH-CR: 56.6 (10.9) vs IR-MPH: 56.8 (11.0) (*p* = NS) *CTRS-R* MPH-CR: 56.3 (10.2) vs IR-MPH: 52.8 (8.5) (*p* < .05)
Jain et al. (2007)	18–60 years *N* = 39	Double-blind, randomized, placebo-controlled 5 weeks/crossover	MPH-CR: 10–80 mg/day Placebo	*CGI-I* MPH-CR: 2.6 (1.0) vs placebo: 3.7 (1.4) (*p* = .005)
Schachar et al. (2008)	6–15 years *N* = 17	Double-blind, randomized, placebo-controlled 2 weeks/crossover	MPH-CR: 1.2 mg/kg/day IR-MPH: 0.6 mg/kg BID Placebo	*IOWA-C* Change vs placebo Overall: MPH-CR: −1.38 (2.27); IR-MPH: 0.66 (2.05) (*p* < .05 vs placebo for both) Time points (1–10 h post-dose): MPH-CR and IR-MPH (*p* < .05 vs placebo)
OROS-MPH				
Wolraich et al. (2001)	6–12 years *N* = 277	Double-blind, randomized, placebo-controlled 4 weeks/parallel group	OROS-MPH: 18, 36, 54 mg/day IR-MPH: 5, 10, 15 mg TID Placebo	*IOWA CTRS, I/O subscale* OROS-MPH: 5.98 (3.91) vs placebo: 9.77 (4.02) (*p* < .05) OROS-MPH: 5.98 (3.91) vs IR-MPH: 6.35 (4.31) (*p* = NS)
Wilens et al. (2006a)	13–18 years *N* = 175	Double-blind, randomized, placebo-controlled 2 weeks/parallel group	OROS-MPH: 18–72 mg/day Placebo	*ADHD-RS-IV total score* OROS-MPH: −14.93 (10.72) vs placebo: −9.58 (9.73) (*p* = .001)
Biederman et al. (2006b)	19–60 years *N* = 141	Double-blind, randomized, placebo-controlled 6 weeks/parallel group	OROS-MPH: 36 mg/day, up to 1.3 mg/kg/day Placebo	*AISRS score by week* OROS-MPH vs placebo (*p* < .05 at weeks 3 to 6)
Swanson et al. (2004) (COMACS Study)	6–12 years *N* = 184	Double-blind, randomized, placebo-controlled 3 weeks/crossover	OROS-MPH: 18, 36, 54 mg/day MPH-CD: 20, 40, 60 mg/day Placebo	*SKAMP-D* OROS-MPH vs placebo: time points (1.5–12.0 h post-dose; *p* < .05) OROS-MPH vs MPH-CD: time point (12 h post-dose; *p* < .05) *SKAMP-D, PERMP-A, PERMP-C* OROS-MPH (36, 54 mg/day) vs placebo: time points (1.5–12.0 h post-dose; *p* < .05)
Lopez et al. (2003)	6–12 years *N* = 36	Single-blind, randomized, placebo-controlled 5 weeks/crossover	OROS-MPH: 18, 36 mg/day MPH-LA: 20 mg/day Placebo	*SKAMP-A, SKAMP-D, SKAMP-Combined, PERMP-C, PERMP-A* MPH-LA vs placebo (*p* < .001) *SKAMP-A. SKAMP-D, SKAMP-Combined* MPH-LA vs OROS-MPH: AUC (0–4 h) change (*p* < .05) MPH-LA vs OROS-MPH: AUC (0–8 h) change (*P* significance varies) *PERMP-A, PERMP-C* MPH-LA vs OROS-MPH: AUC (0–8 h) change (*p* = NS)
Silva et al. (2005)	6–12 years *N* = 54	Single-blind, randomized, placebo-controlled 6 weeks/crossover	OROS-MPH: 18, 36 mg/day MPH-LA: 20, 40 mg/day Placebo	*SKAMP-A* All active treatments: time points (0.5–12 h post-dose; *p* < .038 vs placebo) *SKAMP-D* All OROS-MPH treatments, MPH-LA 40 mg/day: time points (0.5–12 h post-dose; *P* ≤ .003 vs placebo) *Math-Attempted* OROS-MPH: time points (1–8 h, 12 h; *p* < .05 vs placebo) *Math-Correct* OROS-MPH: time points (1, 3, 4, 6, 8 h; *p* < .05 vs placebo)
Medori et al. (2008)	18–63 years *N* = 394	Double-blind, randomized, placebo-controlled 5 weeks/parallel group	OROS-MPH: 18, 36, or 72 mg/day Placebo	*CAARS-O:SV total score* Significantly larger improvement with 18 mg, 36 mg, and 72 mg/day OROS-MPH vs. placebo: mean change from baseline −10.6 (*p* = .01), −11.5 (*p* = .01), and −13.7 (*p* < .001) versus −7.6, respectively
Adler et al. (2009)	18–65 years *N* = 226	Double-blind, randomized, placebo-controlled 7 weeks/parallel group	OROS-MPH: 36, 54, 72, 90, or 108 mg/day Placebo	*AISRS total score (LS mean change from baseline)* OROS-MPH: −10.6 vs placebo: −6.8 (*p* = .012)
Casas et al. (2011)	18–65 years *N* = 279	Double-blind, randomized, placebo-controlled 13 weeks/parallel group	OROS-MPH: 54 or 72 mg/day Placebo	*CAARS-O:SV total score (mean change from baseline)* OROS-MPH 72 mg −15.7 vs placebo −10.4 (*p* = .0024) OROS-MPH 54 mg −12.5 vs placebo −10.4 (NS)
Spencer et al. (2011)	19–60 years *N* = 53	Single-blind, randomized, substitution study 6 weeks/parallel group	Adults who were stable on IR-MPH (TID) were randomized to equipotent doses of OROS-MPH or to continue IR-MPH	*AISRS total score* No significant difference between OROS-MPH and IR-MPH through 6 weeks
Murray et al. (2011) Wigal et al. (2011) Armstrong et al. (2012)	9–12 years Study 1: *N* = 68 Study 2: *N* = 71	Two double-blind, randomized, placebo-controlled, crossover, analog classroom studies	OROS-MPH: 18–54 mg/day Placebo	Pooled data (*N* = 139): Treatment effects on PERMP-A, PERMP-C, SKAMP-A, and SKAMP-D were present at all post-dose assessment points (1, 2, 4, 10, 11, and 12.5 h post-dose; *p* < .0001 for all comparisons vs. placebo)

^a^Values are mean (SD) unless otherwise noted

*ACTRS* Abbreviated Conners’ Teacher Rating Scale, *ADHD* attention-deficit/hyperactivity disorder, *ADHD*-*RS*-*IV* Attention Deficit/Hyperactivity Disorder Rating Scale Version IV, *AISRS* Adult ADHD Investigator Symptom Report Scale, CAARS-O:SV Conners’ Adult ADHD Rating Scale–screening version, *CD* controlled-delivery, *CGI*-*I* Clinical Global Impression-Improvement, *COMACS* a comparison of methylphenidates in an analog classroom setting, *CPRS*-*R* Conner’s Parent Rating Scale-Revised, *CR* controlled-release;* CTRS-R* Conners’ Teacher Rating Scale-Revised, *d*-AMP *d*-amphetamine, *I*/*O* inattention/overactivity, *IOWA*-*C* Inattention/Overactivity With Aggression-Conners’ Scale, *IR* immediate-release, *IOWA* *CTRS* IOWA Conners’ Teacher Rating Scale, *LA* long-acting, *MPH* methylphenidate, *NS* not significant, *OROS* osmotic-release oral system, *PERMP*-*A* permanent product measure of performance-attempted, *PERMP*-*C* PERMP-correct, *SKAMP*-*A* Swanson, Kotkin, Agler, M-Flynn, Pelham Rating Scale-Attention, *SKAMP*-*D* SKAMP-deportment, *SR* sustained-release, *TID* three times daily

**Table 3:** Short-term (≤13-week), randomized, controlled clinical efficacy trials of long-acting amphetamine-based psychostimulants in children and adults with ADHD

Author (year)	Participants (age, *N*)	Duration/design	Treatment (dose)	Primary outcome^a^
MAS-XR				
Spencer et al. (2006b)	13–17 year *N* = 278	Double-blind, randomized, placebo-controlled 4 weeks/parallel group	MAS-XR: 10, 20, 30, 40 mg/day Placebo	*ADHD-RS-IV total score (change from baseline*) MAS-XR 10–40 mg/day: −17.8 vs placebo: −9.4 (*P* ≤ .005)
Biederman et al. (2002)	6–12 year *N* = 563	Double-blind, randomized, placebo-controlled 3 weeks/parallel group	MAS-XR: 10, 20, 30 mg/day Placebo	*CGIS-T (change-average of morning and afternoon assessments)* MAS-XR 10 mg/day: −5.3 vs placebo: −0.9 (*P* ≤ .001) MAS-XR 20 mg/day: −6.0 vs placebo: −0.9 (*P* ≤ .001) MAS-XR 30 mg/day: −6.4 vs placebo: −0.9 (*P* ≤ .001)
Weisler et al. (2006)	>18 years *N* = 248	Double-blind, randomized, placebo-controlled 4 weeks/parallel group	MAS-XR: 20, 40, 60 mg/day Placebo	*ADHD-RS-IV total (placebo-adjusted difference)* MAS-XR 20 mg/day: −6.6 (*P* ≤ .001) MAS-XR 30 mg/day: −7.2 (*P* ≤ .001) MAS-XR 40 mg/day: −7.8 (*P* ≤ .001)
McCracken et al. (2003)	6–12 year *N* = 49	Double-blind, randomized, placebo-controlled 7 weeks/crossover	MAS-XR: 10, 20, 30 mg/day Placebo	*SKAMP-A* MAS-XR 10 mg/day: time points (4.5–7.5 h, 10.5 h; *p* < .05 vs placebo) MAS-XR 20 mg/day: time points (4.5–12 h; *p* < .05 vs placebo) MAS-XR 30 mg/day: time points (1.5–12 h; *p* < .05 vs placebo) *SKAMP-D* MAS-XR 10 mg/day: time points (4.5–9 h; *p* < .05 vs placebo) MAS-XR 20 mg/day: time points (1.5–10.5 h; *p* < .05 vs placebo) MAS-XR 30 mg/day: time points (1.5–12 h; *p* < .05 vs placebo)
*d*-AMP-XR				
James et al. (2001)	7–12 year *N* = 35	Double-blind, randomized, placebo-controlled 8 weeks/crossover	*d*-AMP-XR: 5–30 mg/day *d*-AMP-IR: 5–30 mg/day MAS-IR: 5–30 mg/day Placebo	*CTHS (overall)* *d*-AMP-XR vs placebo (*p* < .001) *d*-AMP-XR vs *d*-AMP-IR (*p* = NS) *d*-AMP-XR vs MAS-IR (*p* = .04) *Actometer and parental rating of behavior:* *d*-AMP-XR vs placebo (*p* = .007) Actometer: *d*-AMP-XR vs placebo: time points (1.75–12 h: *p* < .0007)
Pelham et al. (1990)	8–13 year *N* = 22	Double-blind, randomized, placebo-controlled 8 weeks/crossover	*d*-AMP-XR: 10 mg/day IR-MPH: 10 mg BID MPH-SR: 20 mg/day Pemoline 56.25 mg/day Placebo	*Abbreviated CTRS-Teacher* *d*-AMP-XR vs placebo (*p* < .05) IR-MPH vs placebo (*p* = NS) MPH-SR vs placebo (*p* = NS) Pemoline vs placebo (*p* < .05) *Abbreviated CTRS-Counselor* *d*-AMP-XR vs placebo (*p* < .05) IR-MPH vs placebo (*p* < .05) MPH-SR vs placebo: (*p* < .05) Pemoline vs placebo (*p* = NS) *Behavioral and classroom measures* *d*-AMP-XR vs placebo (*p* < .05 for all behavioral measures) *d*-AMP-XR vs placebo (*p* < .05 for following rules and timed reading; number attempted)
LDX				
Adler et al. (2008)	18–55 years *N* = 414	Double-blind, randomized, placebo-controlled 4 weeks/parallel group	LDX: 30, 50, 70 mg/day Placebo	*ADHD-RS-IV total score (change from baseline)* LDX 30 mg/day: −16.2 (1.06) vs placebo: −8.2 (1.43) (*p* < .0001) LDX 50 mg/day: −17.4 (1.05) vs placebo: −8.2 (1.43); (*p* < .0001) LDX 70 mg/day: −18.6 (1.03) vs placebo: −8.2 (1.43); (*p* < .0001)
Biederman et al. (2007b)	6–12 years *N* = 285	Double-blind, randomized, placebo-controlled 4 weeks/parallel group	LDX: 30, 50, 70 mg/day Placebo	*ADHD-RS-IV total score (change from baseline)* LDX 30, 50, 70 mg/day vs placebo (*p* < .001)
Biederman et al. (2007a)	6–12 years *N* = 50	Double-blind, randomized, placebo-controlled 3 weeks/crossover	LDX: 30, 50, 70 mg/day MAS-XR: 10, 20, 30 mg/day Placebo	*SKAMP-D overall mean score (change from 1st time point)* LDX all doses: 0.8 (0.1) vs placebo: 1.7 (0.1) (*p* < .0001) *SKAMP-D time course* LDX all doses: time points (2–12 h) vs placebo (*p* < .0001) MAS-XR all doses: time points (2–12 h) vs placebo (*p* < .0001) LDX vs MAS-XR (*p* = NS)
Wigal et al. (2009)	6–12 years *N* = 113	Double-blind, randomized, placebo-controlled 2 weeks/crossover	LDX: 30, 50, 70 mg/day Placebo	*SKAMP-D (change from pre-dose)* LDX all doses: time points (1.5–13 h) vs placebo (*p* < .005)
Wigal et al. (2010)	18–55 years *N* = 105	Double-blind, randomized, placebo-controlled 2 weeks/crossover	LDX: 30, 50, 70 mg/day Placebo	*PERMP total (post-dose average)* Difference in LS mean (LDX-placebo): 23.4 (*p* < .0001) *PERMP total time course* (LS mean change from pre-dose) Time points (2–14 h) vs placebo (*p* < .001)
Findling et al. (2011)	13–17 years *N* = 309	Double-blind, randomized, placebo-controlled 4 weeks/parallel group	LDX: 30, 50, or 70 mg/day Placebo	*ADHD-RS-IV total score (LS mean change from baseline)* −18.3, −21.1, −20.7 for 30, 50, and 70 mg/d LDX, respectively; −12.8 for placebo (*p* ≤ .0056 versus placebo for each)

^a^Values are mean (SD) unless otherwise noted

*ADHD* attention-deficit/hyperactivity disorder, *ADHD-RS-IV* Attention Deficit/Hyperactivity Disorder Rating Scale Version IV, *AMP* amphetamine; *CD* controlled-delivery; *CGIS-T* Conners’ Global Index Scale Teacher version, *CTHS* Conners’ Teacher Hyperactivity Scale, *CTRS-R* Conners’ Teacher Rating Scale-Revised; *d-AMP* *d*-amphetamine; *IR* immediate-release, *LDX* lisdexamfetamine dimesylate, *LS* least squares, *MAS* mixed amphetamine salts, *SR* sustained-release, *NS* not significant, *PERMP* Permanent Product Measure of Performance, *SKAMP-A* Swanson, Kotkin, Agler, M-Flynn, and Pelham Rating Scale-Attention, *SKAMP-D* SKAMP-Deportment, *XR* extended-release

### Methylphenidate sustained-release

There are limited clinical trial data of MPH-SR in children with ADHD (Table 2). In a comparison of immediate-release MPH (MPH-IR), with MPH-SR, controlled-release *d*-AMP, and pemoline in boys with ADHD, sustained-release MPH demonstrated efficacy versus placebo in some behavioral measures, some performance-based tasks, and structured assessments by counselors on the Abbreviated Conners’ Teachers Rating Scale (ACTRS), but not on the teacher-rated ACTRS (Pelham et al. 1990). MPH-SR has a duration of effect of approximately 8 h (Novartis Pharmaceuticals Canada Inc.).

### Methylphenidate controlled-release

MPH-CR has shown significant efficacy in reducing ADHD symptoms (Table 2). In children with ADHD, both MPH-CR and IR-MPH yielded similar, statistically significant reductions from baseline in Conners’ Parent Rating Scale-Revised scores (CPRS-R)(Weiss et al. 2007). However, superior symptom reduction with IR-MPH versus MPH-CR was observed based on Conners’ Teacher Rating Scale-Revised (CTRS-R). In adults with ADHD, MPH-CR yielded significantly better Clinical Global Impressions-Improvement (CGI-I) ratings versus placebo after 2 weeks (Jain et al. 2007). Using an analog classroom crossover protocol, Schachar et al. (2008) compared MPH-CR and IR-MPH with placebo in children with ADHD. Significant improvements versus placebo were seen with MPH-CR for up to 10 h post-dosing, based on change from baseline on Inattention/Overactivity With Aggression-Conners’ scale (IOWA-C) overall and subscores for inattention/overactivity and aggression/defiance.

### Osmotic-release oral system methylphenidate

OROS-MPH has demonstrated efficacy in ADHD symptom reduction in children, adolescents, and adults (Table 2). In a 4-week, parallel-group, placebo-controlled study, OROS-MPH was significantly more effective than placebo in children with ADHD, based on endpoint scores for the Inattention/Overactivity subscale of the IOWA Conners’ Teacher Rating Scale (Wolraich et al. 2001). In a 2-week, parallel-group study, OROS-MPH significantly reduced ADHD Rating Scale IV (ADHD-RS-IV) scores in adolescents with ADHD compared with placebo (Wilens et al. 2006a). Similar improvements compared with placebo in the symptoms of ADHD have been described in adults treated with OROS-MPH (Adler et al. 2009; Biederman et al. 2006b; Medori et al. 2008).

In a head-to-head trial, subtle variations of timing and magnitude of symptom control were observed between MPH-CD and OROS-MPH. Although MPH-CD showed greater efficacy in the morning hours, OROS-MPH exhibited longer-lasting efficacy, extending up to 12 h following a single morning dose (Pelham et al. 2001). Head-to-head comparisons of long-acting MPH (MPH-LA) and OROS-MPH in children with ADHD using analog classroom protocols over 8–12 h found that both active treatments improved Permanent Product Measure of Performance (PERMP) math test scores for the number of problems answered correctly (PERMP-C) (Lopez et al. 2003) and Swanson, Kotkin, Agler, M-Flynn, and Pelham Rating Scale (SKAMP)-deportment and SKAMP-Attention scores (Silva et al. 2005). While both treatments were generally effective and well tolerated, superiority of one treatment over the other in such laboratory school settings is dependent on the formulation with the highest expected plasma MPH concentration across the post-dosing period (Swanson et al. 2004). Placebo-controlled analog classroom studies indicate that OROS-MPH has a duration of action of at least 12.5 h (the last time point assessed) in children with ADHD (Armstrong et al. 2012; Murray et al. 2011; Wigal et al. 2011).

### Mixed amphetamine salts extended-release

A number of randomized controlled clinical trials have shown that controlled-release MAS (MAS-CR) is effective versus placebo for reducing ADHD symptoms in children, adolescents, and adults (Table 3) (Biederman et al. 2002; Spencer et al. 2006b; Weisler et al. 2006). An analog classroom trial in children showed that a significant effect of MAS-CR over placebo emerged at 1.5 h post-dosing and was maintained for up to 12 h, based on improvements from baseline at end point in SKAMP-D and math test scores (McCracken et al. 2003).

### Dextroamphetamine controlled-release

Studies of the efficacy of controlled-release AMP (AMP-CR) in participants with ADHD are limited. Pelham et al. compared treatment arms with AMP-CR, IR-MPH, MPH-SR, and pemoline in boys. Although all treatments were superior to placebo in some behavioral measures, only pemoline and AMP-XR were superior to placebo by the teacher-rated ACTRS. The duration of efficacy was characterized as within 2 h of ingestion and up to 9 h post-dose (Pelham et al. 1990). In an analog classroom trial of AMP-CR in children, objective actometer measures and parent ratings of behavior were improved compared with placebo from 1.75 to 12 h following a single morning dose. The effects of AMP-CR were “less robust” than those of IR-MAS in the morning hours after dosing but were extended for 3–6 h longer (James et al. 2001).

### Lisdexamfetamine dimesylate

The efficacy of LDX compared with placebo in reducing the symptoms of ADHD has been demonstrated in patients across the lifespan (Table 3). In 4-week trials of LDX 30, 50, and 70 mg in children, adolescents, and adults with ADHD, all doses of LDX demonstrated significant improvements in ADHD-RS-IV scores compared with placebo (Adler et al. 2008; Biederman et al. 2007b; Findling et al. 2011). Mean rates of response (defined as >30 % improvement in ADHD-RS-IV scores and CGI-I ratings of much improved or very much improved) were approximately 80 % at end point in children treated with LDX 70 mg compared with less than 20 % for placebo (Biederman et al. 2007b). Furthermore, improvements compared to placebo in CPRS-R scores in children with ADHD were maintained until 6 pm following an early morning dose (Biederman et al. 2007b). In an analog classroom study in children with ADHD, the therapeutic effects of LDX extended from 1.5 to 13 h post-dose (the first and last time points assessed) based on improvements in SKAMP and PERMP scores (Wigal et al. 2009). In a simulated adult workplace environment study, the therapeutic effects of LDX were maintained from 2 to 14 h post-dose (the first and last time points measured) in adults with ADHD, as shown by improvements in PERMP scores versus placebo (Wigal et al. 2010). The demonstration that the efficacy of LDX is maintained for at least 13 h in children and 14 h in adults suggests that this prodrug may be the longest-acting stimulant treatment for ADHD.

### Meta-analyses of the effectiveness of long-acting stimulants

Meta-analyses have compared efficacy outcomes from multiple studies of different stimulant formulations (Faraone 2009, 2012; Faraone and Glatt 2010). In an analysis of 32 trials of 16 medications in youths with ADHD, the mean effect size for long-acting stimulants was 0.95 compared with 0.99 for immediate-release stimulants (Faraone 2009). Similarly, in 19 trials of 13 ADHD drugs in adults, mean effect sizes were 0.73 and 0.96 for long-acting and immediate-release stimulants, respectively (Faraone and Glatt 2010). A meta-analysis of efficacy studies in children with ADHD, based only on ADHD Rating Scale and Clinical Global Impressions outcomes, found that a pooled effect size for LDX of approximately 1.5 was significantly (*p* < 0.001) greater than the pooled effect size of the other medications (Faraone 2012). In adult studies, LDX effect sizes were similar to those of other medications. Using numbers-needed-to-treat (NNT) to compare the efficacy of stimulants medications across 23 clinical trials in children and adolescents, NNT (95 % confidence intervals) were slightly lower (i.e., fewer patients were required to see a positive effect) for formulations of AMP (2.0 [1.7, 2.2]) than MPH (2.6 [2.4, 2.8]), although mean NNT values were not calculated for long-acting and immediate-release formulations (Faraone et al. 2006).

## Safety of psychostimulants

Short- and long-acting psychostimulants share similar adverse event profiles (Banaschewski et al. 2006). Adverse events most commonly associated with the use of psychostimulants to treat ADHD include neurological (headache, dizziness, insomnia, seizures), psychiatric (mood/anxiety, tics, psychosis), and gastrointestinal (abdominal pain, poor appetite leading to weight loss/slowed growth) effects. In general, these events are mild and/or temporary (Graham et al. 2011). Areas of particular concern in the use of psychostimulants to treat ADHD include their effects on growth and cardiovascular parameters, and their potential for abuse.

### Effect on weight and growth

An analysis of 20 longitudinal studies found that long-term psychostimulant use in children with ADHD resulted in statistically significant delays in growth versus age-related norms (Faraone et al. 2008). Effects appeared to be dose-related, were more apparent for weight than height, were similar between MPH and AMP formulations, and, in many cases, appeared to normalize over time despite continued treatment (Faraone et al. 2008). In the MTA (Multimodal Treatment Study of Children with ADHD), the largest longitudinal study of children with ADHD, average relative size of patient (a composite of height and weight as *z* scores) was negatively related to the average cumulative exposure to psychostimulants. Growth slowing for newly medicated versus untreated participants was found for the first 14 months, was attenuated at 24 months, and was non-significant at 36 months (Murray et al. 2008; Swanson et al. 2007). Nevertheless, it is recommended that height and weight are monitored in patients receiving stimulant medications, including long-acting formulations.

### Cardiovascular events

Cardiovascular safety concerns with psychostimulant ADHD medications were raised based on rare occurrences of sudden death and other cardiac events (Vetter et al. 2008). Psychostimulants modulate cardiovascular contractility and heart rate via sympathomimetic effects (Wilens et al. 2006b), and changes in vital signs have been noted with MPH and AMP treatments, including increases in systolic and diastolic blood pressure (~2–6 mmHg) and heart rate (~8 beats per minute) (Wilens et al. 2005). In clinical trials of psychostimulants in children and adults, no clinically significant changes in atrial or ventricular conduction or repolarization have been observed (Wilens et al. 2006b). An elevated risk of cardiac-related emergency visits with current psychostimulant use versus non-use has been reported (Winterstein et al. 2007; Winterstein et al. 2009), but in the majority of healthy children with no history of or current cardiovascular abnormalities, psychostimulants produced cardiovascular effects of minimal clinical significance (Findling et al. 2001, 2005; Safer 1992; Wilens et al. 2006b). Similarly, a large, retrospective, population-based cohort study concluded that ADHD medication use in children and young and middle-aged adults was not associated with an increased risk of serious cardiovascular events compared with non-use (Cooper et al. 2011; Habel et al. 2011). However, when treatment with any psychostimulant formulation is contemplated in a patient with structural heart disease, or in a patient who has a personal or a family history of syncope or sudden death, respectively, a pediatric or cardiologic consultation prior to ADHD pharmacological treatment is strongly advised (Graham et al. 2011).

### Abuse, misuse, and diversion

Prescription stimulants are classified as controlled substances, and their abuse, misuse, and diversion are important public health and safety concerns (Kollins 2008; Wilens et al. 2008). Since euphoria and drug “liking” are linked to a more rapid rate of absorption and delivery to the brain (Volkow and Swanson 2003), it follows that controlling the rate of stimulant release may modify its potential for abuse. Support for a lower abuse potential for long-acting compared with short-acting stimulants includes greater subjective responses for immediate-release stimulants than OROS-MPH in healthy adults (Parasrampuria et al. 2007; Spencer et al. 2006a). The formulation of stimulants as once-daily medications also reduces the likelihood of diversion by removing the requirement for drug administration at school.

Many long-acting stimulants can be manipulated to facilitate more rapid absorption of the active ingredient, for example by crushing or dissolving the medications in order to facilitate intranasal or parenteral administration (Mao et al. 2011). However, the physical characteristics of stimulant formulations, such as the non-deformable shell of the OROS-MPH capsule, may make them more difficult to break, cut, or crush. The prodrug design of LDX means that the rate of active *d*-AMP release is limited by the rate of enzymatic conversion, regardless of the route of drug administration or capsule intactness. Thus, the plasma concentration–time profile of intranasal LDX in healthy men is similar to that following oral administration (Ermer et al. 2011). In individuals with a history of stimulant abuse, scores in the Drug Rating Questionnaire-Subject liking scale for oral LDX (≤100 mg) were no different to placebo, whereas the equivalent oral dose of *d*-AMP (40 mg) was favored over placebo (Jasinski and Krishnan 2009a). Similarly, unlike intravenous *d*-AMP 20 mg, an equivalent intravenous dose of LDX (50 mg) did not produce subjective abuse-related liking scores (Jasinski and Krishnan 2009a, b).

## Adherence

Non-adherence to medication for chronic illnesses is estimated to be approximately 50 % (World Health Organization 2003). In ADHD, the prevalence of medication discontinuation or non-adherence is reported to range from 13 to 64 % (Adler and Nierenberg 2010). After 14 months of treatment with MPH in the MTA study, analysis of saliva samples indicated that only 53.5 % of patients were adherent at every assay point and that almost 25 % of patients were non-adherent at 50 % or more of their assays (Pappadopulos et al. 2009). Studies that have examined the impact of formulation on adherence and persistence of stimulant medications for ADHD include several retrospective claims analyses (Christensen et al. 2010; Marcus et al. 2005; Sanchez et al. 2005). The largest of these identified over 60,000 newly treated patients with ADHD (Christensen et al. 2010). This analysis indicated that the mean (SD) adherence (defined as the ratio of the number of days therapy supplied to the total number of days persistent) to long-acting stimulants (0.56 [0.32]) was significantly greater (*p* < 0.0001) than that for short- (0.43 [0.35]) and intermediate-acting (0.47 [0.35]) stimulants. Similarly, the mean (SD) persistence (defined as the number of days out of 365 days plus the index day that the patient remained on their index therapy) on long-acting stimulants (239.5 [145.8]) was significantly greater (*p* < 0.0001) than for short- (186.7 [154.8]) or intermediate-acting (185.6 [153.4]) stimulants (Christensen et al. 2010). Furthermore, a chart review of Spanish adults with ADHD found that the switch from short-acting MPH to long-acting MPH was associated with a significant improvement in all items of the Simplified Medication Adherence Questionnaire (Ramos-Quiroga et al. 2008). These data suggest that the choice of formulation has important consequences on the adherence and persistence of ADHD stimulant medications.

## Treatment individualization

Evidence-based ADHD guidelines recognize that medication strategies should be tailored for the individual. Among the factors to consider when selecting an appropriate medication for a patient with ADHD are drug class and the formulation required to give the desired pharmacokinetic and pharmacodynamic profiles. The choice of medications for ADHD includes both stimulants and non-stimulants. With regard to stimulants, although mean responses to MPH and AMP are similar, individuals may respond differently to the two drugs. As reviewed by Arnold (2000), approximately 28 % will respond preferentially to AMP, 17 % will respond preferentially to MPH, and less than 13 % will not respond to either (Arnold 2000). Where stimulant medications are indicated, the selection of formulation should be based both on clinical requirements and on the preferences of the patient and/or their family. Short- and long-acting formulations provide treatment options ranging from approximately 4 h to, in the case of LDX, more than 13 h in children (Wigal et al. 2009) and more than 14 h in adults (Wigal et al. 2010). It is important to note that suboptimal response to one class or formulation of stimulant does not predict failure of another. Thus, a recent post hoc analysis found that the clinical effectiveness and rates of remission in children with ADHD who were treated with LDX were similar in patients with a previous suboptimal response to MPH treatment to those of the overall study population (Jain et al. 2011).

## Conclusions

The development of controlled-release formulations of stimulants and the stimulant prodrug LDX has greatly increased the number of pharmacological treatment options for patients with ADHD. At equivalent systemic exposures, the efficacy and safety of long-acting stimulants appear to be equivalent to short-acting formulations. However, long-acting medications offer several potential benefits to patients. Long-acting stimulants offer efficacy for at least 13–14 h without augmentation. The convenience of once-daily dosing may contribute to improved adherence of long-acting stimulants compared with short-acting stimulants. Prodrug technology may also provide lower inter- and intra-patient variability in exposure than mechanical controlled-release systems. Furthermore, long-acting stimulants may be less prone to abuse than their short-acting counterparts. Thus, the development of long-acting formulations of stimulants provides important additional treatment options for the management of ADHD.
